# Assembled and annotated reference genome and demographic history of the eastern chipmunk (*Tamias striatus*)

**DOI:** 10.1007/s00438-026-02427-x

**Published:** 2026-05-02

**Authors:** Scarlet A. Shifflett, Esther Weyer, Madolyn L. MacDonald, Vincenzo A. Ellis

**Affiliations:** 1 Department of Entomology and Wildlife Ecology, University of Delaware, Newark, DE USA; 2 Center for Bioinformatics and Computational Biology, University of Delaware, Newark, DE USA; 3 Bioinformatics Data Science Core, University of Delaware, Newark, USA DE

**Keywords:** Comparative genomics, Evolution, *Tamias*, Positive selection, MSMC2

## Abstract

The eastern chipmunk (*Tamias striatus*) is an abundant North American sciurid rodent and the only extant member of the subgenus *Tamias*. Despite its abundance, a reference genome has not been produced for this species. Here we present an assembled and annotated reference genome for the eastern chipmunk using PacBio HiFi long read sequencing data. We sequenced the genome of a vouchered male eastern chipmunk collected in Maine and stored at the National Museum. We compared the eastern chipmunk’s genome with the genome of the Siberian chipmunk (*Tamias sibiricus*; subgenus *Eutamias*), the only other species in the *Tamias* genus with an annotated reference genome. The genomes were similar with 55.45% of genes showing collinearity and chromosomal rearrangements only occurring between chromosomes 4 and 8. We identified 219 genes under positive selection in the eastern chipmunk relative to the Siberian chipmunk. A coalescent analysis inferred the effective population size of the Maine eastern chipmunk may have steadily decreased through the Pleistocene epoch, consistent with a reduction in available habitat. We also generated short-read Illumina sequencing data from two additional eastern chipmunks collected in New Castle County, Delaware and Carbon County, Pennsylvania. We calculated nucleotide diversity over 10 kb windows across the genome (mean = 0.0014 ± 0.0012 s.d.) and identified genes with high nucleotide diversity. The eastern chipmunk reference genome will facilitate future population genetic and evolutionary studies including those investigating its role as a reservoir host for the bacterium that causes Lyme disease.

**Supplementary Information:**

The online version contains supplementary material available at 10.1007/s00438-026-02427-x.

## Introduction

High quality, species specific, reference genomes can improve conspecific read mapping, facilitate accurate variant calling, and may increase the reliability of various population genetic measures (Akopyan et al. 2025). Furthermore, reference genomes allow for population level estimates of demographic history (Li and Durbin 2011) and a genome wide perspective in selective pressures driving species evolution. As sequencing costs decrease and computational technologies improve, it is becoming easier to obtain high quality whole genome references for non-model organisms (Surget-Groba and Montoya-Burgos 2010). Using genomic approaches to study natural populations of non-model organisms is important for advancing evolutionary and ecological research, and applied research into conservation and disease management (Ekblom and Galindo 2011; Blanchong et al. 2016; Brandies et al. 2019). Nevertheless, many common and well-studied organisms do not have reference genomes available.

**Fig. 1:**
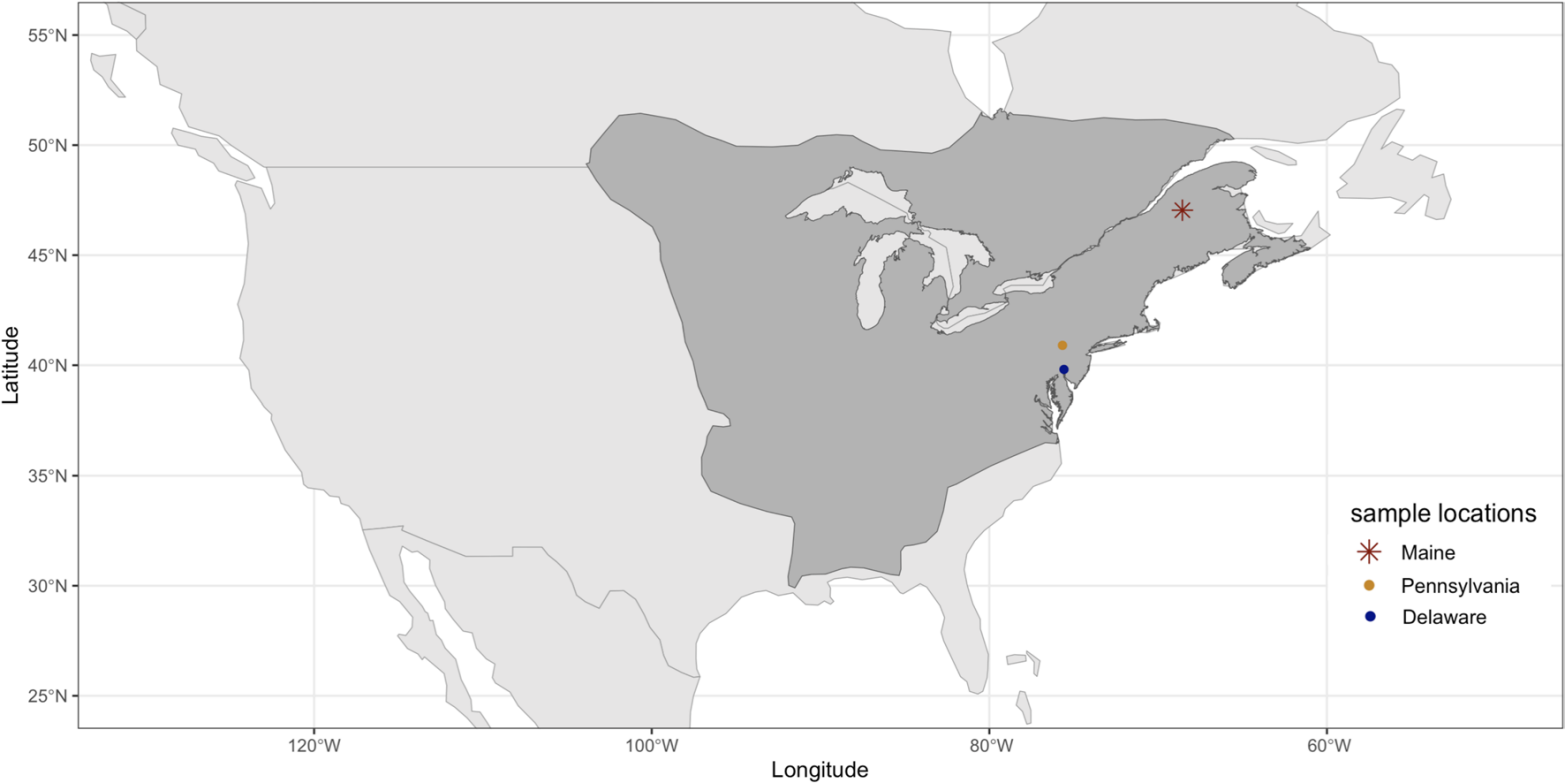
Species distribution map of the eastern chipmunk (*Tamias* *striatus*); species range is shown in dark gray and colored points show sampling locations for the three individuals used in this study. The red star shows the location of the chipmunk sampled in Maine and used for the reference genome assembly. The Pennsylvania and Delaware chipmunks, representing the additional short-read sequencing data, are shown as an orange and blue dot, respectively. This figure was created with the 2016 IUCN Red List *Tamias striatus* assessment version 6.3 (IUCN 2016) shape file using R v.4.2.2 and the following R packages: sf v1.0.16 (Pebesma, Edzer 2018 AD; Pebesma and Bivand 2023), rnaturalearth v1.2.0.9000 (Massicotte and South 2026), rnaturalearthdata v.1.0.0 (South et al. 2024), and ggplot2 v3.5.2 (Wickham 2016)

The eastern chipmunk (*Tamias striatus*) is a small rodent in the family Sciuridae and the only extant species in the subgenus *Tamias*. The genus *Tamias* also includes two other subgenera; *Neotamias* with 23 species distributed across western North America and *Eutamias* represented only by the Siberian chipmunk (*T. sibiricus*; Snyder 1982; Solari and Baker 2007). The eastern chipmunk is mostly ground dwelling and occupies deciduous forests from the southern United States to Canada (Snyder 1982; Fig. 1). The northeastern distribution of the species strongly coincides with human cases of Lyme disease, the most common vector-borne disease in the United States (Kugeler et al. 2021). Eastern chipmunks are known to be competent reservoir hosts for the Lyme disease bacterium *B. burgdorferi* and play an important role in maintaining the prevalence and diversity of the bacterium within an ecosystem (LoGiudice et al. 2003; Brisson and Dykhuizen 2004; Ostfeld et al. 2014). *B. burgdorferi* infections are more genetically diverse in eastern chipmunks than expected by chance (Shifflett et al. 2023) and a single allele (called allele “U”) of the pathogen’s outer surface protein *C* gene appears restricted to infections of eastern chipmunks (Brisson and Dykhuizen 2004; Shifflett et al. 2023). Eastern chipmunks have been shown to be of interest in other disease systems including human babesiosis (Hersh et al. 2012), other *Borrelia spp*. (Siy et al. 2021), and have been experimentally shown to be a potential amplifying host of West Nile Virus (Platt et al. 2007).

Despite the commonness of eastern chipmunks across their range and their role in a variety of disease systems, our ecological and evolutionary understanding of the species has been limited to microsatellite genotyping and mitochondrial DNA analyses (Piaggio and Spicer 2000; Rowe et al. 2006; Sarver et al. 2016; St-Hilaire et al. 2017; Vandal et al. 2020). While informative, microsatellite loci by themselves are limited in their ability to be applied in certain population genetic analyses including estimating effective population sizes, obtaining high confidence diversity measures, and understanding higher level systemic relationships (Putman and Carbone 2014; Zimmerman et al. 2020). Furthermore, the use of mitochondrial DNA alone can reduce the ability to fully resolve species level phylogeographic patterns, population demographic histories, and phylogenies of closely related species (Egger et al. 2007; Godinho et al. 2008; Yannic et al. 2010; Sarver et al. 2016; Dallaire et al. 2024), highlighting the importance of a genomic approach. By incorporating an organism’s complete set of DNA, genomic approaches give a more thorough understanding of the ecological and evolutionary history of a species. We can continue to advance species level genomic approaches through the addition of reference genomes. Currently there is no reference genome for the eastern chipmunk. The Siberian chipmunk is the only assembled and annotated *Tamias* genome on NCBI (Li et al. 2022), with the Siberian and eastern chipmunk having an estimated divergence time of 9.64 Mya (Upham and Esselstyn 2019).

To address this issue, we assembled and annotated a contig level genome of the eastern chipmunk (*T. striatus*) using HiFi PacBio long-reads from a museum vouchered specimen collected in Maine. We compared the genome with that of its closest *Tamias* relative with an annotated reference genome, the Siberian chipmunk, and conducted tests for positive selection. With the addition of our genome to the sciurid group, we present a phylogeny of all sciurid species with an NCBI annotated reference genome. We also used the eastern chipmunk genome to examine the species’ historical demography. Finally, we generated Illumina short-read data from two additional eastern chipmunks to produce an initial assessment of nucleotide diversity across the chipmunk genome.

## Methods

### Sample collection and sequencing

We used tissue from a vouchered eastern chipmunk specimen (*Tamias striatus lysteri*) housed at The National Museum of Natural History (Washington DC, USA). This adult male specimen was collected in Aroostook County, Maine (47.04186, −68.56389; Fig. 1) in September of 2019. Collected tissue was stored in 95% ethanol and kept in a −80 °C freezer. The voucher specimen can be found at the National Museum of Natural History under the catalog number USNM 603667. The University of Delaware DNA Sequencing and Genotyping Center performed high molecular weight DNA extraction from the tissue using the Qiagen MagAttract HMW DNA kit. Post extraction, DNA was quantified with a Qubit Fluorimeter (Thermo Fisher Scientific, Waltham, MA, USA) and DNA fragment sizes were assessed using a Femto Pulse system (Agilent, Santa Clara, CA, USA). DNA was sheared into 14 kb fragments using Megaruptor3 followed by library preparation via the PacBio HiFi SMRTbell protocol using SMRTbell Express Template Prep Kit 3.0 (PacBio, 102–182–700). DNA fragments smaller than 8 kb were removed using a Blue Pippin instrument (Sage Science, Beverly, MA, USA) with a final average library DNA fragment size of 14 kb. DNA was then sequenced on one SMRT cell using the PacBio Revio with the Revio binding and sequencing kit and the 30-h movie option.

We used a Tomahawk live trap to capture an adult male eastern chipmunk in northern Delaware (39.808184, −75.578589) in April 2023. A 2 mm sterile ear punch (Fisherbrand Animal Ear Punch) was used to take an ear biopsy from the individual. The tissue was immediately stored in RNA later (Thermo Fisher Scientific, Waltham, MA, USA) and transferred to 4 °C for 48 h before storing in a −20 °C freezer until DNA extraction. Live trapping and tissue collection was done under the State of Delaware Scientific Collecting Permit No. 2023-WSC-016 and the University of Delaware’s Institutional Animal Care and Use Committee protocol AUP No. 1377. We also collected a 2 mm ear biopsy from a museum vouchered specimen (unknown sex; voucher 22,057) housed at the Academy of Natural Sciences in Philadelphia, PA. The specimen was collected in Carbon County, PA on October 13th, 1974. Sampling localities are shown in Fig. 1.

We used the standard DNeasy Blood and Tissue kit (Qiagen, Germantown, MD, USA) tissue protocol to extract DNA from the live capture ear biopsy. DNA was extracted from the museum specimen tissue using a modified protocol with the same kit. Briefly, within a sterile biosafety cabinet, we placed the museum ear biopsy in 300 µl ultrapure water for 3 h at room temperature, followed by a quick centrifugation, and removed the remaining liquid. We repeated this step with fresh ultrapure water and then proceeded with the standard tissue extraction protocol from the DNEasy Blood and Tissue kit, with the exception of using 30 µl of Proteinase K instead of the recommended 20 µl to improve protein digestion and DNA yield of the low-quality skin tissue. We eluted the DNA with ultrapure water in the final step of the protocol. Extracted DNA was sent to the University of Delaware DNA Sequencing and Genotyping Center for library preparation and short-read sequencing on an Illumina NextSeq 2000. The Revvity Rapid DNA-Seq 2.0 kit (Revvity, Waltham, MA, USA) was used for library preparation for the Pennsylvania sample following option one in the manufacturer’s manual; no fragmentation was performed prior to library preparation as input DNA was of sufficient size. Library amplification included 1 ng of input DNA and 15 PCR cycles followed by an additional 0.8 × magnetic bead cleanup to remove adapter dimers. Library preparation for the Delaware chipmunk used the QuantaBio sparQ DNA Frag and Library prep kit (QuantaBio, Beverly, MA, USA) following the manufacturer’s protocol with 1 ng of DNA input. DNA was fragmented for 25 min followed by library amplification with a 13 cycle. Both the Pennsylvania and Delaware samples were sequenced on the Illumina Nextseq 2000 using a P2 kit with 2 × 109 reads. The raw short-reads have been submitted to NCBI under the BioProject accession PRJNA1372205.

### Genome assembly and annotation

We performed quality control of the HiFi PacBio reads using NanoPlot v.1.43.0 (De Coster and Rademakers 2023). Reads were not filtered due to the high quality of the raw PacBio reads. The genome was assembled using a variety of de novo assemblers including Flye v.2.9 (Kolmogorov et al. 2019), hifiasm v.0.19.5 (Cheng et al. 2021), and PacBio’s SMRT Link software v.13.0.0 (https://www.pacb.com/smrt-link/). We accessed genome assembly quality using QUAST v.5.1.0 (Mikheenko et al. 2018) and BUSCO v.5.4.7 (Manni et al. 2021) with hifiasm providing the best assembly (Supplementary Table 1). BUSCO was run using the “glires_odb10” lineage, which consisted of 13,798 single-copy orthologs representing Rodentia and lagomorph species. We also checked the assembly for contamination by querying the contigs against the core_nt database v.1.1 using BLAST v.2.15.0 (Altschul et al. 1990; Camacho et al. 2009).

Genome annotation was completed in four general steps. First, we identified repeats in the assembly using RepeatModeler v.2.0.3 (Flynn et al. 2020) and RepeatMasker v.4.1.2 (Smit et al. 2013). The coordinates of the complex repeats were extracted from the RepeatMasker results and given as input to the Maker v.3.01.04 annotation pipeline (Cantarel et al. 2008; Campbell et al. 2014) for hard-masking. For the second step, we performed an initial evidence based annotation round using Maker (Cantarel et al. 2008) with canonical Marmotini (protein sequences downloaded from UniProtKB release 2024_5; Apweiler 2004) and transcripts from the Siberian chipmunk (Li et al. 2022). Next, Braker v.3.0.8 (Hoff et al. 2019) was used for gene model training using Sciuridae proteins from UniProtKB release 2024_6 and mapped Siberian chipmunk RNA reads downloaded from NCBI SRA (SRR4249994; Ma et al. 2016). Our final step involved a second round of Maker with the Braker GFF file provided directly to Maker.

We assigned putative function to the annotated genes following Maker Support Protocols 2 and 3 (Campbell et al. 2014). Functional domains and gene ontology (GO) terms were assigned via Interproscan v.5.53–87.0 (Blum et al. 2021) following steps 4 and 5 of Basic Protocol 5 (Campbell et al. 2014). The annotated genome was filtered to only include proteins with a predicted function, removing 4,089 identical isoforms, and keeping only the longest isoform per gene. We identified and annotated the mitochondrial genome using MitoFinder v.1.4.2 (Allio et al. 2020) and used circularMT v.1.0.0 (Goodman and Carr 2024) for visualization. The raw long-reads, and the assembled genome have been submitted to NCBI under the BioProject accession PRJNA1372205. The assembled genome can also be found in Figshare alongside the annotated genome at 10.6084/m9.figshare.c.8332780.

### Population genetics

We removed Illumina adapters and trimmed the short-read sequencing data from the Delaware and Pennsylvania eastern chipmunks for quality control using Trim Galore v.0.6.10 (Krueger 2007). Trimmed forward and reverse reads were mapped to the assembled eastern chipmunk reference genome using BWA mem v.0.7.17 (H. Li and R. Durbin 2009). Minimap2 v.2.22 (Li 2018, 2021) was used to map the PacBio long-read data from the Maine individual back to its own reference genome. Minimap2 keeps secondary mapping alignments within the output bam file; we used samtools v.1.19.1 (Danecek et al. 2021) to remove all secondary alignments before proceeding with variant calling. Pandepth v.2.26 (Yu et al. 2024) was used to calculate coverage percentage of mapped reads on each contig along with the average read depth for all samples. All other mapping statistics were obtained with samtools v.1.19.1 stats and flagstat commands (Danecek et al. 2021).

Unmapped reads were saved as FASTQ files using samtools (Danecek et al. 2021). The reads were mapped to the *B. burgdorferi* reference genome using BWA mem for short-read sequencing samples and minimap2 for the one long-read sequencing sample to confirm that the chipmunks were not infected with *B. burgdorferi*, a common chipmunk pathogen.

PCR duplicates were marked for all mapped files using Picard v.3.4.0 (Broad Institute, GitHub Repository 2019) before calling variants using GATK v.4.3.0 HaplotypeCaller (Van Der Auwera and O’Connor 2020). We used the -ERC GVCF flag in HaplotypeCaller to produce intermediate VCF files for each sample. GATK GenotypeGVCFs was used to combine the intermediate VCF files and call the final joint genotype VCF file. We filtered the joint VCF file to remove all indels and keep variants with a minimum quality score of 30 and a minimum read depth of 6X. VCF filtering was done using VCFtools v.0.1.16 (Danecek et al. 2011).

We estimated nucleotide diversity (Nei and Li 1979) across 10 kb windows and observed heterozygosity for each individual using VCFtools (Danecek et al. 2011) with the filtered joint VCF file. Nucleotide diversity was visualized along the genome using ggplot2 v.3.5.2 (Wickham 2016) in R v.4.2.2 (R Core Team 2020). We identified outlier windows as those within the top 1% of all calculated diversities; these windows represented the most extreme nucleotide diversity values within the genome. We also determined if those windows occurred within or contained annotated genes with *Mus musculus* homologs. We then used those identified genes and the *Mus musculus* reference genome as the input for the DAVID Bioinformatics Resources v.2025_1 (hereafter DAVID) functional annotation table using all default annotation categories (Huang et al. 2009; Sherman et al. 2022). The functional annotation table lists specific functions for each gene for a known reference species. *Mus musculus* was used because it is the closest model organism to the eastern chipmunk with well-studied gene functions.

We used the Multiple Sequentially Markovian Coalescent model 2 (MSMC2) (Malaspinas et al. 2016; Schiffels and Wang 2020) to examine the demographic history with the single diploid reference genome of the Maine eastern chipmunk. Following the protocol in Schiffels and Wang (2020), we made a reference genome mappability mask using a k-mer length of 35 bp and a stringency value of 0.5. This mappability mask was used to produce the final contig level masked bed file using MSMC2’s program makeMappabilityMask.py. Next, VCF and masked bed files were generated for each contig using the PacBio sequencing reads and assembled reference genome. When calling genotypes, we used a minimum mapping and base quality score of 30 and skipped indels. MSMC2’s program bamCaller.py was used within this step to generate the masked bed files. The masked reference genome bed file, the bed file generated through variant calling, and the corresponding VCF file were used to generate the MSMC2 input with the program generate_multihetsep.py. This step produced an output file with four columns representing the contig name, variant site position, the number of called sites since the previous variant site, and the parental haplotype (in our case this value was always two). Of the 400 contigs, 211 passed all filtering steps and were used in the MSMC2 analysis. MSMC2 was first run with the default time interval parameters -p 1*2 + 25*1 + 1*2 + 1*3. The default time intervals resulted in a final graph typical of an overfitted model, with an extremely high effective population size followed by a rapid decrease. To correct model overfitting, we used fewer time intervals and combined more ancient time intervals. Our final parameters were 1*2 + 10*1 + 1*8. We obtained confidence intervals around the estimated coalescence rates by artificially generating bootstrapped data via MSMC2’s program multihetsep_bootstrap.py. Using a mutation rate of 2.0 × 10^–9^ per site per year, we converted the scaled time to years and the estimated coalescence rates and effective population sizes. This mutation rate has been used in previous demographic history analyses of species within the sciurid group (Gossmann et al. 2019; Wolf et al. 2022) and produce the same results when we tested the average mammalian mutation rate of 2.2 × 10^–9^ (Kumar and Subramanian 2002). Even when incorrectly estimating mutation rates and generation times for sequentially Markovian coalescent based models, the final shape of the plot should not change (Mather et al. 2020). Data were visualized in R v.4.2.2 (R Core Team 2020) using the R package ggplot2 v.3.5.2 (Wickham 2016).

### Comparative genomics

We compared our contig-level assembled genome of the eastern chipmunk to the chromosome-level assembly of the Siberian Chipmunk (Li et al. 2022), the closest related organism with an annotated reference genome. We used MCScanX v.1.0.0 (Wang et al. 2012) to look at the genomic synteny between the eastern and Siberian chipmunk based on gene annotations. Our MCScanX parameters included eastern chipmunk assembled contigs greater than 10 million base pairs, having at least 10 genes in a collinear block and having a match score of 250. OrthoVenn3 Web v.2.0.0 (Sun et al. 2023) was used to identify shared and unique orthologs between the eastern and Siberian chipmunk annotations. Orthovenn3 also carries out a GO enrichment analysis (Ashburner et al. 2000; The Gene Ontology Consortium et al. 2023) on orthologs unique to the eastern chipmunk annotated genome compared to the background list of orthologs shared between the two species.

We tested genes for positive selection across the eastern chipmunk genome relative to the Siberian chipmunk genome using codeml in PAML v.4.10.9 (Yang 2007; Álvarez-Carretero et al. 2023). The eastern gray squirrel (*Sciurus carolinensis*) and Eurasian red squirrel (*Sciurus vulgaris*) genomes were used as phylogenetic outgroups (Mead et al. 2020b, a). To test for positive selection, we first found orthologous genes among the four species using their corresponding amino acid fasta gene files with Orthofinder v.2.5.5 (Emms and Kelly 2015, 2019). We filtered Orthofinder’s output to only include orthogroups that contained one gene per species and had all four species represented. MAFFT v.7.450 (Katoh and Standley 2013) was used to create multiple sequence alignment (MSA) files for each single copy ortholog sequence fasta file. Orthogroup MSA files were used in PhyKIT v.2.0.3 (Steenwyk et al. 2021) to create a concatenated matrix of all orthologous genes. PhyKIT’s output was used in RAxML v.8.2.12 (Stamatakis 2014) to create a multi-gene species tree. RAxML included an initial pre-filter step to remove orthogroup MSA files with alignment errors.

We then used PAL2NAL v.14.1 (Suyama et al. 2006) to get a codon-based DNA alignment file from the amino acid ortholog MSA file and its corresponding nucleotide MSA. Our PAL2NAL output was used as the input to PAML’s codeml. We used codeml’s branch-site model to calculate nonsynonymous to synonymous mutation ratios (dN/dS, also known as omega) to test for codon site level positive selection on the eastern chipmunk’s species tree branch. When omega is greater than one, the higher ratio of nonsynonymous to synonymous mutations is consistent with positive selection (Yang and Bielawski 2000). Codeml control file specifications are seen in Supplementary Fig. 1. The same species tree was used for each orthogroup (Supplementary Fig. 2). Genes with a significant likelihood ratio test (LRT) statistic (after false discovery adjustment) and at least one codon site with a 95% posterior probability (via codeml’s Bayes Empirical Bayes analysis) of having an omega value greater than one were kept for downstream analysis.

We used DAVID v.2025_1 (Huang et al. 2009; Sherman et al. 2022) to perform an enrichment analysis to determine if any genes under positive selection had overrepresented functional annotations compared to a list of background genes. The input gene list for DAVID consisted of the *Mus musculus* homolog for each gene found to be under positive selection with the background list of genes represented with all of the functionally annotated genes in the *Mus musculus* genome. Background genes represent a list of all genes that had the possibility of being selected from the original analysis (positive selection). Briefly, DAVID takes the input list of genes under positive selection and converts them to DAVID ID terms. Genes with DAVID IDs are then given an associated functional annotation term with multiple genes typically being associated with one function (functional annotation chart). For each functional term, DAVID uses a Fisher’s exact test to determine if the number of input genes within the given functional term are overrepresented compared to the number of background genes within the same functional term. DAVID reports the Fisher’s exact test statistic along with the *P*-value and its Benjamini-adjusted *P*-value. To reduce the redundancy of functional annotation terms, DAVID groups terms into larger biologically relevant clusters (functional annotation clustering). The Fisher’s exact *P-*values for each functional annotation term are carried over to the functional clusters. DAVID produces an enrichment score equivalent to the -log of the mean of *P-*values within a cluster. Enrichment scores are positively related to the degree of overrepresentation of functional terms within the cluster. We filtered DAVID’s functional categories to include direct biological processes, direct molecular functions, and annotations from UniProtKB (Apweiler 2004) with biological processes and molecular function as keywords.

We utilized our newly assembled genome to infer the eastern chipmunk’s phylogenetic relationship to 10 additional sciurid species. We selected sciurid species with an annotated reference genome and a corresponding genome level coding sequence fasta file. These criteria gave us 11 species, nine in the ground squirrel subfamily Xerinae (arctic ground squirrel (*Urocitellus parryii*), Alpine marmot (*Marmota marmota marmota*), woodchuck (*Marmota monax*), golden-mantled ground squirrel (*Callospermophilus lateralis*), yellow-bellied marmot (*Marmota flaviventris*), thirteen-lined ground squirrel (*Ictidomys tridecemlineatus*), Siberian chipmunk (*Tamias sibiricus*), and the eastern chipmunk *(Tamias striatus*)) and two species in the tree squirrel subfamily Sciurinae (eastern gray squirrel (*Sciurus carolinensis*) and the Eurasian red squirrel (*Sciurus vulgaris*)). The American beaver (*Castor* *canadensis*) was used as an outgroup in the sciurid phylogeny. We ran Orthofinder v.2.5.5 (Emms and Kelly 2015, 2019) on all 11 CDS fasta files to find single copy orthologous genes. MSA files were created for each orthologous gene using MAFFT v.7.450 (Katoh and Standley 2013); downstream analysis included MSA files containing sequence information for all 12 species. We created gene level trees with RAxML’s v.8.2.12 maximum likelihood inference using the model setting GTRGAMMA and the ‘-f d’ rapid hill-climbing algorithm (Stamatakis 2014). Gene level trees were used in ASTRAL v.5.7.8 (Mirarab et al. 2014) to estimate a species tree with quartet support annotations (-t 1). ASTRAL’s species tree was visualized with PearTree v.0.2.2 (Rambaut 2026).

## Results

### Genome assembly and annotation

**Fig. 2:**
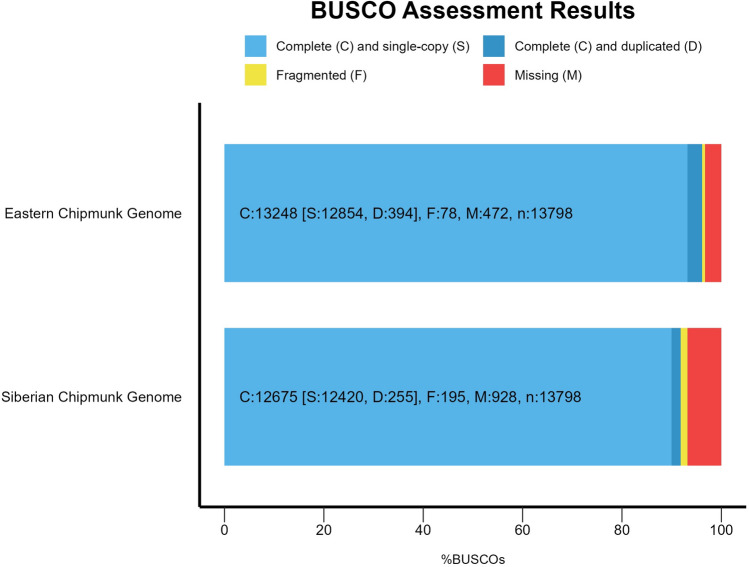
BUSCO assessment results showing the quantitative assessment of the assembled eastern chipmunk (*Tamias striatus*) genome using hifiasm compared to the Siberian chipmunk (*T. sibiricus*) assembled genome. The Siberian chipmunk reference genome was assembled in Li et al. 2022. Our newly assembled eastern chipmunk reference genome has a 96.1% completion score compared to the 91.8% completion score for the Siberian chipmunk. BUSCO v.5.4.7 was used to create this figure

Hifiasm gave the most contiguous genome assembly with 431 contigs (N50 = 87,501,790), compared to 728 contigs with PacBio’s smrtlink software (N50 = 41,531,070), and 15,439 contigs with flye (N50 = 489,677); we present the hifiasm assembly here. The largest contig was 169,491,321 bp and the number of unknown bases (Ns) was zero. The final assembled genome had a GC (%) of 40.43 and is 2.5 Gb in size compared to the 2.59 Gb genome of the Siberian Chipmunk (Li et al. 2022). BUSCO assessment results showed that the hifiasm assembled genome was of high quality and completeness with 96.1% of ortholog sequences complete, 0.6% fragmented, and 3.3% missing; 93.2% of the complete sequences were single copies and 2.9% were duplicate copies (Fig. 2).

The final annotation contained 27,784 proteins with a protein BUSCO showing high completeness of 94.4% complete single copy ortholog proteins (92.1% single copy and 2.3% duplicated). Fragmented proteins made up 1.8% and 3.8% of proteins were missing. Annotation statistics for the assembled and annotated genome (without isoforms and the mitochondrial genome) are presented in Table 1.

**Table 1:** Genome annotation statistics for the assembled and annotated eastern chipmunk reference genome. Statistics were calculated from the GFF file without isoforms and without the mitochondrial genome

Number of genes/mRNSAs/proteins	27,784
Number of exons	211,323
Number of introns	183,136
Number of single exon genes	5189
Mean exons per mRNA	7.6
mean introns per mRNA	6.6
Total gene length	786,330,428
Mean gene length	28,301
Longest gene	497,045
Shortest gene	69
Protein BUSCO Score	C:94.4%[S:92.1%,D:2.3%],F:1.8%,M:3.8%

We visually compared our annotated mitochondrial genome with a published eastern chipmunk mitochondrial genome (Supplementary Fig. 3; GenBank Accession Number NC_032375.1, Sarver et al. 2016). Our final mitochondrial genome assembly is 16,697 bp, only slightly longer than the published assembly (16,533 bp).

We mapped Illumina short read data from the two eastern chipmunks to our newly assembled eastern chipmunk reference genome with 98.62% and 99.68% of reads mapping from the live-captured Delaware chipmunk and the museum specimen from Pennsylvania, respectively. As expected, extracted DNA from the museum specimen sample was fragmented due to a lack of tissue preservation; the average, filtered sequence length of the museum specimen was 88.4 bp ± 0.001 s.e., compared to 111.5 bp ± 0.001 s.e. for the live-caught Delaware chipmunk. Furthermore, 99.97% of the PacBio long-read data (average sequence length of 13,751.5 bp ± 1.525 s.e.) mapped back to the newly assembled reference genome. The percentage of mapped reads per contig along with the average read depth for each sample can be found in Supplementary Fig. 4.

### Population genetics

The average genome-level nucleotide diversity was 0.0014 ± 0.0012 s.d. and ranged from 6.7e^−6^ to 0.03 among 10 kb windows of the genome. Of the 267,163 genome windows, 268 had nucleotide diversities greater 0.0094 (representing the top 1% of all calculated diversities; Supplementary Fig. 5), and 104 windows occurred within 49 annotated genes having *M. musculus* homologs. The list of 49 M*. musculus* homolog genes resulted in 45 DAVID IDs with functional annotation records per DAVID’s functional annotation analysis (Supplementary Table 2). Functional annotations included genes involved in olfaction and pheromone response, angiogenesis, and immune system processes. Observed heterozygosity (proportion of sequenced sites that were heterozygous) was 0.393 for the Delaware chipmunk (n sites = 10,034,799), 0.412 for the Maine chipmunk (n sites = 14,170,327), and 0.555 for the Pennsylvania chipmunk (n sites = 6,150,560).

**Fig. 3:**
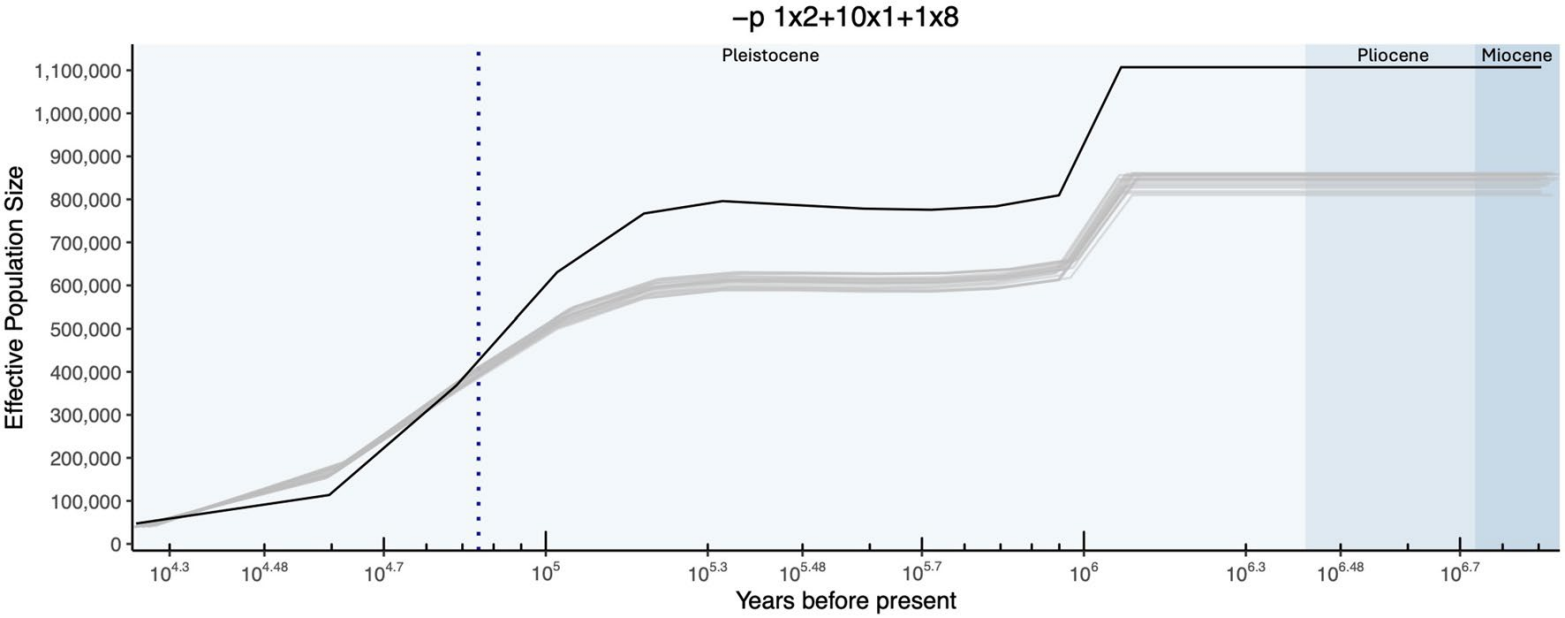
MSMC2 demographic history of the eastern chipmunk from one diploid genome from Maine. Estimated effective population size is shown on the y-axis and log scale time in years before present is shown on the x-axis. Population demographic history of the Maine individual is shown in black with bootstrapped runs shown in gray. Geological epochs are indicated by blue backgrounds (from right to left, Miocene, Pliocene, and Pleistocene). The blue vertical dashed line represents the start of when Maine is predicted to have been covered by the Laurentide ice sheets, with the deglaciation process in Maine not occurring within the graph’s data limits (Dalton et al. 2022). This figure was created with R v.4.2.2 and the ggplot2 v3.5.2 package

The MSMC2 software revealed a steady decrease in effective population size from the Pliocene through the Pleistocene epochs (Fig. 3) of the eastern chipmunk sampled from Maine. The general shape of the plot stayed the same when testing different time interval parameters. This is also true of our 20 MSMC2 runs using artificial genomes assembled from bootstrapped sequencing data (Fig. 3). While the bootstrapped data showed the same trend as the real data, they had lower effective population size estimates in more ancient times and slightly higher estimates in more recent times. We therefore focus on the temporal trend in effective population size rather than claiming specific effective population sizes.

### Comparative genomics

**Fig. 4:**
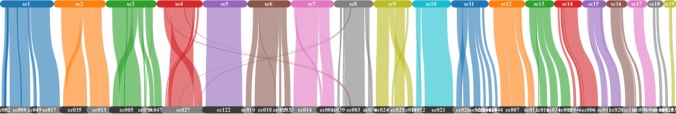
Genomic synteny between the Siberian chipmunk chromosome level assembly and eastern chipmunk contig level assembly. Siberian chipmunk chromosome IDs are shown on top with sc1-sc19. Eastern chipmunk contigs are shown at the bottom and only include the 45 contigs with more than 10 million base pairs. Gene blocks per chromosome are represented with unique colors. MCScanX v.1.0.0 was used to create this figure

**Fig. 5:**
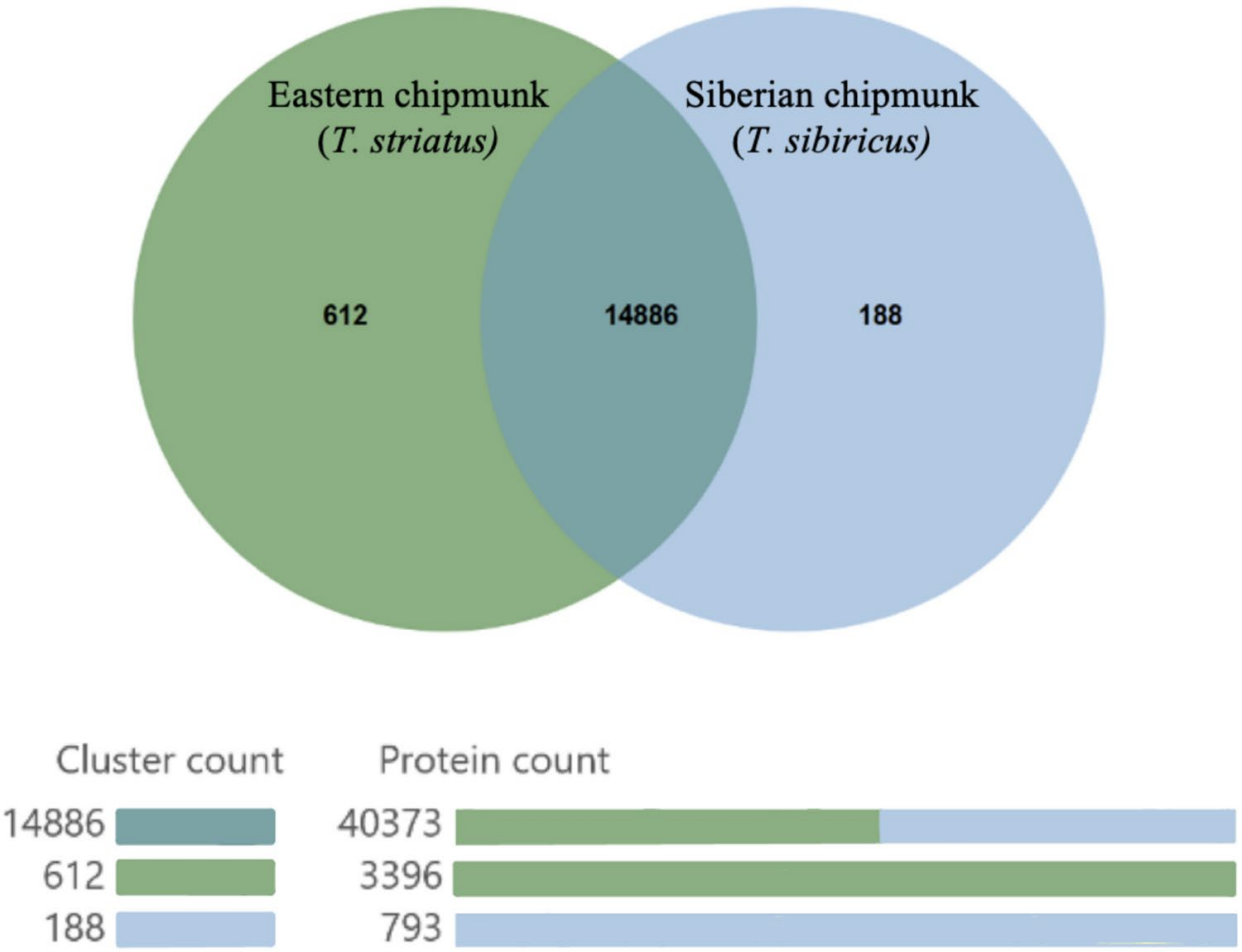
Visual comparison of shared and unique orthologous clusters for the eastern chipmunk and Siberian chipmunk reference genome. There are 14,886 orthologs and 40,373 proteins shared between the two species. There are 612 orthologs and 3,369 proteins to be unique to the eastern chipmunk genome annotation. OrthoVenn3 was used to create this figure

Genomic synteny between the Siberian chipmunk chromosome-level genome assembly and the eastern chipmunk contig-level genome assembly showed 29,428 collinear genes out of 53,071 total genes (55.45% collinearity; Fig. 4). Despite general concordance in gene synteny between the two *Tamias* species, we identified rearrangements between chromosome 4 and chromosome 8 in the Siberian chipmunk (contigs ec027 and ec003 in the eastern chipmunk; Fig. 4). We found 3,396 proteins unique to the eastern chipmunk and 798 unique to the Siberian chipmunk with 40,373 proteins shared between the species. The eastern and Siberian chipmunk shared 14,886 ortholog clusters; 612 ortholog clusters were unique to the eastern chipmunk and 188 clusters unique to the Siberian chipmunk (Fig. 5). We found 283 enriched GO terms unique to the eastern chipmunk genome relative to the Siberian chipmunk genome (Supplementary Table 3). Of the 283 enriched GO terms, 13 GO terms (175 total genes) were involved in immune related processes. It is important to note that these orthologs may appear to be unique to the eastern chipmunk genome simply because they were missing in the Siberian chipmunk annotation; alternatively, they could reflect differences in evolutionary pressure between the species.

To more fully understand differences in selective pressure between the eastern chipmunk and Siberian chipmunk we calculated dN/dS ratios to quantify positive selection. We first were able to assign 87.4% of genes to 21,854 orthogroups. Of those 21,854 orthogroups, 14,509 had all four species represented and 5,236 orthogroups had single-copy genes. After RAxML’s initial alignment error filtering, we were left with 4,096 orthogroups. Orthogroups that did not pass the RAxML filter contained two or more species with identical sequences. We used the RAxML multi-gene species tree (Supplementary Fig. 2) in all positive selection analyses. PAL2NAL resulted in 3,712 orthogroups MSA files in PAML format used for codeml’s branch-site model of positive selection. 89 of the 3,712 orthogroups branch-site models favored the alternative hypothesis of positive selection on the eastern chipmunk as indicated by a significant likelihood ratio statistic (Supplementary Table 4). Further filtering (Bayes Empirical Bayes posterior probability 95% or greater) resulted in 68 genes under positive selection in the eastern chipmunk (Supplementary Table 5) with 64 of those genes having *Mus musculus* homologs (Supplementary Table 6)*.*

Of the 64 genes, 61 genes had DAVID IDs and an associated functional annotation. Functional annotations included processes related to spermatogenesis, the immune system, blood vessel formation, and transcription activity (Supplementary Table 7). DAVID’s functional annotation chart grouped the 61 genes into 20 functional terms. No terms were considered significantly overrepresented compared to the background set of genes per a Fisher’s exact test (Supplementary Table 8). Of the 20 terms, three terms involved protein binding processes (a direct molecular function) including protein binding (21 genes, accounting for 34.4% of all 61 genes), identical protein binding (9 genes, 14.8%), and 14–3-3 protein binding (2 genes, 3.3%). Functional annotation terms with direct biological processes included genes related to vision and visual perception processes, phosphorylation, differentiation, cartilage development, and the related chondrogenesis.

Redundant functional annotations were further reduced into larger more biologically relevant functional annotation clusters, decreasing the 20 annotation terms into 3 clusters (Supplementary Table 9). Cluster one contained genes related to vision, visual perception, and sensory transduction. Processes related to differentiation, cell differentiation, and developmental proteins were grouped into cluster two. Genes within cluster two included those related to spermatogenesis and one gene related to immune system functioning within the broader terms. The final annotation cluster, Cluster 3, included terms related to DNA binding, transcription, and RNA polymerase processes.

**Fig. 6:**
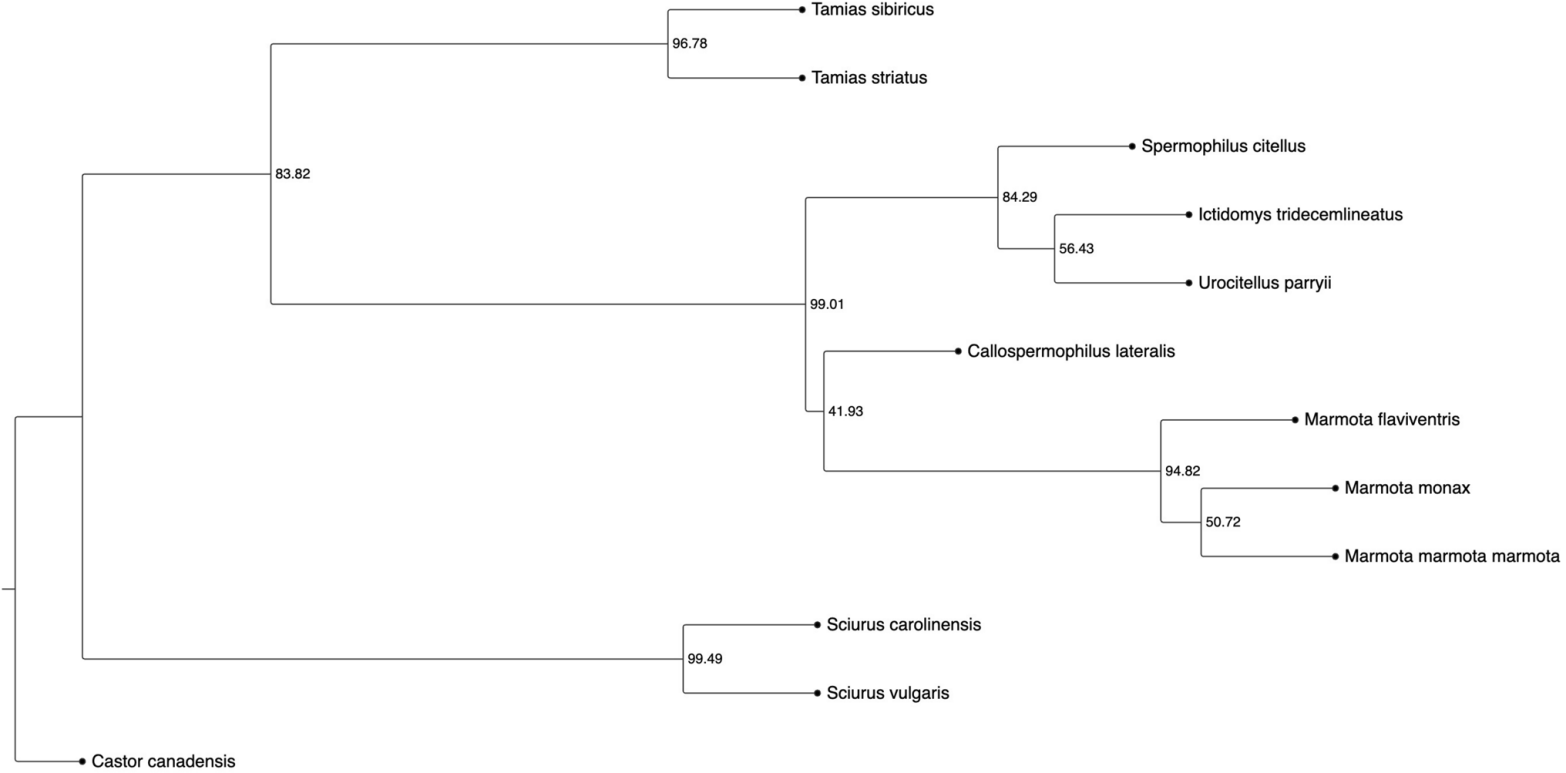
Estimated sciurid phylogeny using 393 nucleotide gene trees for 11 sciurid species, and one species outgroup (American beaver, *Castor canadensis*). The species are as follows: eastern chipmunk (*Tamias striatus*), Siberian chipmunk (*Tamias sibiricus*), European ground squirrel (*Spermophilus citellus*), thirteen-lined ground squirrel (*Ictidomys tridecemlineatus*), arctic ground squirrel (*Urocitellus parryii*), golden-mantled ground squirrel (*Callospermophilus lateralis*), yellow-bellied marmot (*Marmota flaviventris*), woodchuck (*Marmota monax*), alpine marmot (*Marmota marmota marmota*), eastern gray squirrel (*Sciurus carolinensis*), European red squirrel (*Sciurus vulgaris*). Node labels represent the percentage of gene tree quartets supporting the main branch topology. This figure was made using PearTree v.0.2.2

We estimated a species tree of 11 sciurid species, and one outgroup species, from 393 gene trees produced from orthologous nucleotide coding sequences (Fig. 6). Across the 12 species, 81.7% of the CDS regions were assigned to a total 30,660 orthogroups via Orthofinder. Of the 30,660 orthogroups, 447 were single copy ortholog sequences and 393 single copy orthologs had sequences for all 12 species. As expected, the tree separates species within the ground squirrel subfamily from those in the tree squirrel subfamily (represented only by the eastern gray squirrel and European red squirrel).

## Discussion

Here we present a high-quality annotated reference genome of the eastern chipmunk, the only extant species in the subgenus *Tamias*. The final annotated genome contained 27,784 proteins and shared 14,886 ortholog clusters with the Siberian chipmunk (Fig. 5) and generally had concordant synteny with the Siberian chipmunk genome (Fig. 4). Using these data, we determined that the effective population size of the eastern chipmunk sampled in Maine showed a steady decrease from the Pliocene through the Pleistocene epochs (Fig. 3). The eastern chipmunk had several thousand proteins that were not found in the Siberian chipmunk genome. An enrichment analysis with proteins unique to the eastern chipmunk showed 175 genes across 13 GO terms to be involved in immune related processes. We identified 61 genes under positive selection in the eastern chipmunk with known *Mus musculus* homologs. Among the genes under positive selection with direct biological processes, were those related to spermatogenesis, vision, angiogenesis, immunity, DNA binding, transcription, and cell differentiation (Supplementary Tables 6 and 7). Finally, we conducted an initial quantification of nucleotide diversity across the eastern chipmunk genome using samples from Delaware, Pennsylvania, and Maine and found that the windows with highest nucleotide diversity overlapped with genes involved in the olfaction, angiogenesis, and immune related processes.

We estimated the demographic history of the Maine eastern chipmunk using MSMC2 (Schiffels and Wang 2020). Our results show a decrease in effective population size throughout the majority of the Pleistocene epoch (Fig. 3). Effective population size represents the genetic size of a population. It was originally defined as the size of an ideal, neutrally evolving population that undergoes genetic drift at the rate of the census population (Wright 1931, 1933, 1969; Crow and Kimura 1970). Historical census population sizes cannot be estimated for the majority of species; we can, however, estimate historical effective population sizes using genetic data. Effective population size gives us the opportunity to understand historical population trends and infer measures of inbreeding, genetic, drift, and a population’s ability to adapt to changing environments. MSMC2, and similar PSMC (Li and Durbin 2011) based methods, have been used for these exact reasons for many organisms including turtles (Hilgers et al. 2025), flycatchers (Nadachowska‐Brzyska et al. 2016), sea ducks (Cádiz et al. 2024), woolly mammoths (Palkopoulou et al. 2015), Steller’s sea cows (Sharko et al. 2021), and alpine marmots (Gossmann et al. 2019).

We found evidence of a stable effective population size from the late Miocene through the early Pleistocene. This trend cannot be supported with fossil records as the North American chipmunk fossil record is almost nonexistent during the late Miocene through the Pliocene (between 4–9 mya; Jameson 1999). The late Miocene Epoch (around 9.64 to 10 mya) is thought to be the time when the eastern chipmunk diverged from the western chipmunk lineage after the *Tamias* group crossed the Bering land bridge and separated from the Siberian chipmunk in Asia (Hopkins 1967; Ellis and Maxson 1979; Upham and Esselstyn 2019). However, there has been disagreement as to whether chipmunks crossed into North America from Asia (Moore 1961; Nadler et al. 1969; Ellis and Maxson 1979) or vise vera (Black 1972). Multiple crossing events have also been suggested (Jameson 1999) along with the hypothesis that the ancestral stock resided within the Bering land bridge and eventually spread, and speciated, across both sides of the bridge (Levenson et al. 1985).

As the graph moves into the Pleistocene, we start to see a decrease in the estimated effective population size followed by a relatively stable period until around 152.2 kya. It is unclear if this negative slope represents a true decrease in effective population size or signifies a loss in genetic diversity from a population bottleneck and founder effect as the species dispersed across North America. The continuous decrease in effective population size from 75 kya into the final data point around 17.33 kya corresponds to the time period where Maine, and much of the surrounding area, was covered by the Laurentide ice sheets (Dalton et al. 2022). Our results may therefore reflect the genetic consequences of habitat loss during these glacial cycles for this population. The alpine marmot showed a similar decrease in the effective population size throughout the Pleistocene, with modern populations still harboring the consequences with low levels of genetic diversity (Gossmann et al. 2019). We cannot make any strong conclusions about genetic diversity in relation to our demographic history trend due to our extremely low sample size.

Previous mitochondrial data has shown a population expansion of three eastern chipmunk clades after the Last Glacial Maximum (Rowe et al. 2006). Rowe et al. (2006) found the northeastern clade to have the most recent expansion estimated at less than 10 kya compared to the expansion estimates for the western and central clades between 12 and 70 kya. A later expansion of the northeastern eastern chipmunk clade is supported by our low, and non-expanding, effective population size from 70 kya through 17 kya. The Laurentide ice sheets are also estimated to have begun the deglaciation process around 10–14 kya in Maine (Dyke and Prest 1987), further supporting the late expansion of the eastern clade population and our low effective population estimates. It is important to note that PSMC based methods are limited in their inability to distinguish low effective population estimates and population declines from population substructure (Mazet et al. 2015; Bansal and Nichols 2025). We therefore cannot conclude if a true population decrease occurred, or rather numerous historical population subdivisions as the species dispersed. For future refinement of this analysis, additional eastern chipmunk genomes can be incorporated to better estimate more recent demographic changes.

Using the GO enrichment analyses, we found 283 of the 612 unique eastern chipmunk orthologs to be overrepresented in terms of biologic function compared to the background list of orthologs shared between the eastern and Siberian chipmunk. Of those functionally overrepresented orthologs, 13 orthologs were related to immune system processes (Supplementary Table 3). Genes involved in immune system processes were also shown in additional analyses including 12 genes with a high nucleotide diversity values and three genes shown to be under positive selection in the eastern chipmunk compared to the Siberian chipmunk. Both chipmunk species are considered reservoir hosts for their respective geographic groups of bacteria responsible for human Lyme disease cases; eastern chipmunks harbor the North American *Borrelia burgdorferi *sensu stricto and Siberian chipmunks harbor the European and Asian *B. burgdorferi *sensu stricto, *B. afzelii*, and *B. garinii*. However, the eastern chipmunk plays a much larger role in maintaining the *Borrelia* bacterium within the ecosystem (LoGiudice et al. 2003; Brisson and Dykhuizen 2004; Ostfeld et al. 2014) compared to the Siberian chipmunk; the latter being infrequently infected in its native range (Chu et al. 2006). Nevertheless, Siberian chipmunks appear to play a larger role in the Lyme disease system where they are invasive (Marsot et al. 2011, 2013), but are still considered less competent than other hosts of *Borrelia* (Bonnet et al. 2015). For example, one month post *B. afzelii* and *B. burgdorferi* infection, Siberian chipmunks were unable to transmit the bacteria to feeding ticks. In comparison, eastern chipmunks infected with *B. burgdorferi* can infect feeding ticks four months post-infection (McLean et al. 1993).

We used the dN/dS approach to identify 61 genes under positive selection, including genes related to immunity, spermatogenesis, vision, and a variety of regulatory processes (transcription, cell differentiation, DNA binding, and angiogenesis). We found five positively selected genes involved in spermatogenesis and one gene involved in biological processes within the sperm midpiece (Supplementary Table 7). Omega (dN/dS) values for the five spermatogenesis genes and sperm midpiece genes ranged from 133.81 to 999, indicating strong positive selection. Two spermatogenesis genes clustered into the broader categories of cell differentiation, differentiation, and developmental proteins (Supplementary Table 9). Spermatogenesis genes also appeared within an ortholog shown to be unique to the eastern chipmunks compared to Siberian chipmunks from the GO enrichment analysis above (Supplementary Table 3). The rapid evolution of mammalian spermatogenesis and gene expression changes within testes (Brawand et al. 2011; Cardoso-Moreira et al. 2019; Murat et al. 2023) may drive the abundance of spermatogenesis processes shown to be enriched and or under positive selection in the eastern chipmunk compared to the Siberian chipmunk.

Genes with protein binding molecular functions commonly appeared throughout all analyses. Two genes involved in protein binding were shown to be overrepresented in the eastern chipmunk compared to the Siberian chipmunk via the GO enrichment analysis. Of the genes under positive selection in the eastern chipmunk, 34 genes had a protein binding functional term including genes related to spermatogenesis, collagen, and miosis initiation. Two positively selected genes were also included in the 14–3-3 protein binding term and nine genes under identical protein bindings. While, protein binding was common under the molecular function annotation term, the term itself is general and does not provide information on the specific proteins genes are binding to. One gene under positive selection, Lrp2 binding protein (Lrp2bp), identified the protein within the protein binding function (Lrp2). DAVID shows Lrp2 to have a variety of direct biological processes including but not limited to, kidney development, male gonad development, neural tube closer, and sensory perception of sound. Regions of the eastern chipmunk genome having the most extreme nucleotide diversity values included genes involved angiogenesis, the cell cycle, lipid transportation, olfaction and pheromone responses, and various immune system processes.

We estimated a sciurid phylogeny using genome-scale data from the 11 sciurid species with an annotated reference genome at the time of this study (Fig. 6). This phylogeny serves to add a genome-wide perspective to previously published sciurid phylogenies (Steppan et al. 2004; Herron et al. 2004; Sheets and Chavez 2020) and highlight the limited number of species within the Sciuridae family having genome-scale coding sequence data. Our phylogeny shows suboptimal branch support for the arctic ground squirrel and thirteen-lined ground squirrel split, the Woodchuck and Alpine marmot branch, and the branch between the golden-mantled ground squirrel and *Marmota* spp. group. Branch support for these nodes would likely improve with additional species data. The current 11 sciurid species, across 7 genera, with an NCBI annotated reference genome represent only a small portion of the estimated 278 species and 51 genera within the Sciuridae family (Thorington and Hoffmann 2005). To move forward with a genome-scale sciurid phylogeny, and improve upon our presented preliminary phylogeny, annotated reference genomes are needed for the vast majority of sciurid species.

In this paper, we have examined the historical demography and evolutionary history of the eastern chipmunk using the presented data and demonstrated the usefulness of the reference genome to population genetic studies including whole genome sequencing from a 50-year-old museum specimen. The annotated eastern chipmunk reference genome presented here will facilitate evolutionary and ecological studies of this species, the *Tamias* genus, and the greater Sciuridae family.

## Supplementary Information

Below is the link to the electronic supplementary material.

Supplementary file1 (DOCX 10678 KB)

Supplementary file2 (XLSX 64 KB)

## Data Availability

PacBio and Illumina raw sequencing reads are deposited on GenBank under the BioProject PRJNA1372205. The assembled and annotated reference genome are also published on Figshare at 10.6084/m9.figshare.c.8332780.
