# Supplementary material for: Assembled and annotated reference genome and demographic history of the eastern chipmunk (Tamias striatus)
Source: Mol Genet Genomics. 2026 May 2;301(1):106. doi: 10.1007/s00438-026-02427-x (PMC13135541; doi:10.1007/s00438-026-02427-x)
Supplement: Supplementary file 1 — Supplementary file1 (DOCX 10678 KB) [file 438_2026_2427_MOESM1_ESM.docx]

**Supplementary Fig. 1** PAML’s codeml positive selection control file specifications for the branch-site model. The top control file shows the specification for the null model, and the bottom control file shows the alternative model. The control files for both models specify the input ‘seqtype’ as protein-coding DNA sequences (PAL2NAL output; see methods for details), one gene per input file (‘ndata’), and the branch-site model (model = 2 AND NSites = 2). Both model control files also specify the mutation-selection model (CodonFreq = 7) which will estimate model parameters using the observed codon frequencies within the data. The null model does not estimate omega (dN/dS ratio) and uses fix_omega = 0 to calculate the likelihood of a neutral evolution model. The null model also specifies a fixed omega value of one to represent no selective pressure. The alternative model specifies the use of omega estimation with fix_omega = 1 and sets the initial omega value to 1.2 to represent an omega ration under positive selection.

**Supplementary Fig. 2** Species level tree created with RAxML model `-m PROTGAMMAAUTO`. Input sequences included a matrix of all MAFFT aligned ortholog sequence files. Branch support was assessed with a bootstrap analysis consisting of 200 replicates. The output tree file including branch lengths, which were later removed to use as the input tree for the codeml positive selection analyses. This tree was visualized using *Geneious version 2025.2 created by Biomatters. Available from* [*https://www.geneious.com*](https://www.geneious.com/)*.*

**Supplementary Fig. 3** Visual comparison of the presented eastern chipmunk mitochondrial genome (left) with the current reference mitochondrial genome found on GenBank (right). Our assembled mitochondrial genome, identified as ptg000342I, is 16,697 bp long and seen on the left. The current reference mitochondrial genome (NC_032375.1) for the eastern chipmunk is 16,533 bp long and shown on the right. CircularMT v.1.0.0 was used to create this image.

**Supplementary Fig. 4** Mapping statistics from the individual chipmunks from Maine (PacBio long read sequencing data), Delaware (Illumina short read sequencing), and Pennsylvania (Illumina short read sequencing) mapped to the presented eastern chipmunk reference genome. Mapping statistics, shown as the percentage of mapped reads covering each contig (light blue) and the mean read depth per contig (dark blue), were calculated with Pandepth v.2.26 (Yu et al. 2024). A horizontal red line marks the 6x read coverage cut off used in downstream analyses. To avoid overlapping, not all contig bars are labeled.

**Supplementary Fig. 5** Eastern chipmunk nucleotide diversity (pi) across a 10kb window. Pi was calculated using the joint VCF file containing SNP information for the Maine, Delaware, and Pennsylvania chipmunk samples using VCFtools (Danecek et al. 2011). Graphs are separated by contig with and filtered to only show contigs containing outlier pi windows. Pi is shown on the Y-axis with base pair position shown on the X-axis. Each dot represents a 10kb window. Pink dots show outlier windows with a pi value greater than 0.0094, and gray dots represent background windows with pi values less than 0.0094.
